# Supplementary material for: The Human T-cell Leukemia Virus capsid protein is a potential drug target
Source: Nat Commun. 2025 Dec 4;16:10892. doi: 10.1038/s41467-025-65899-2 (PMC12678768; doi:10.1038/s41467-025-65899-2)
Supplement: Supplementary file 1 — Supplementary Information [file 41467_2025_65899_MOESM1_ESM.pdf]

Supplementary Information

The Human T-cell Leukemia Virus capsid protein is a potential drug target

Ruijie Yu[1,2], Prabhjeet Phalora[1,2], Nan Li[1,2], Till Böcking[1,2], David Anthony Jacques[1,2, ✉]

[1]Department of Molecular Medicine, School of Biomedical Sciences, University of New South Wales, Sydney, New South Wales, Australia.
[2]EMBL Australia Node in Single Molecule Science, School of Biomedical Sciences, University of New South Wales, Sydney, New South Wales, Australia.

This file includes the Supplementary Figures 1 to 8 and the Supplementary Tables 1 and 2.

Supplementary Figures

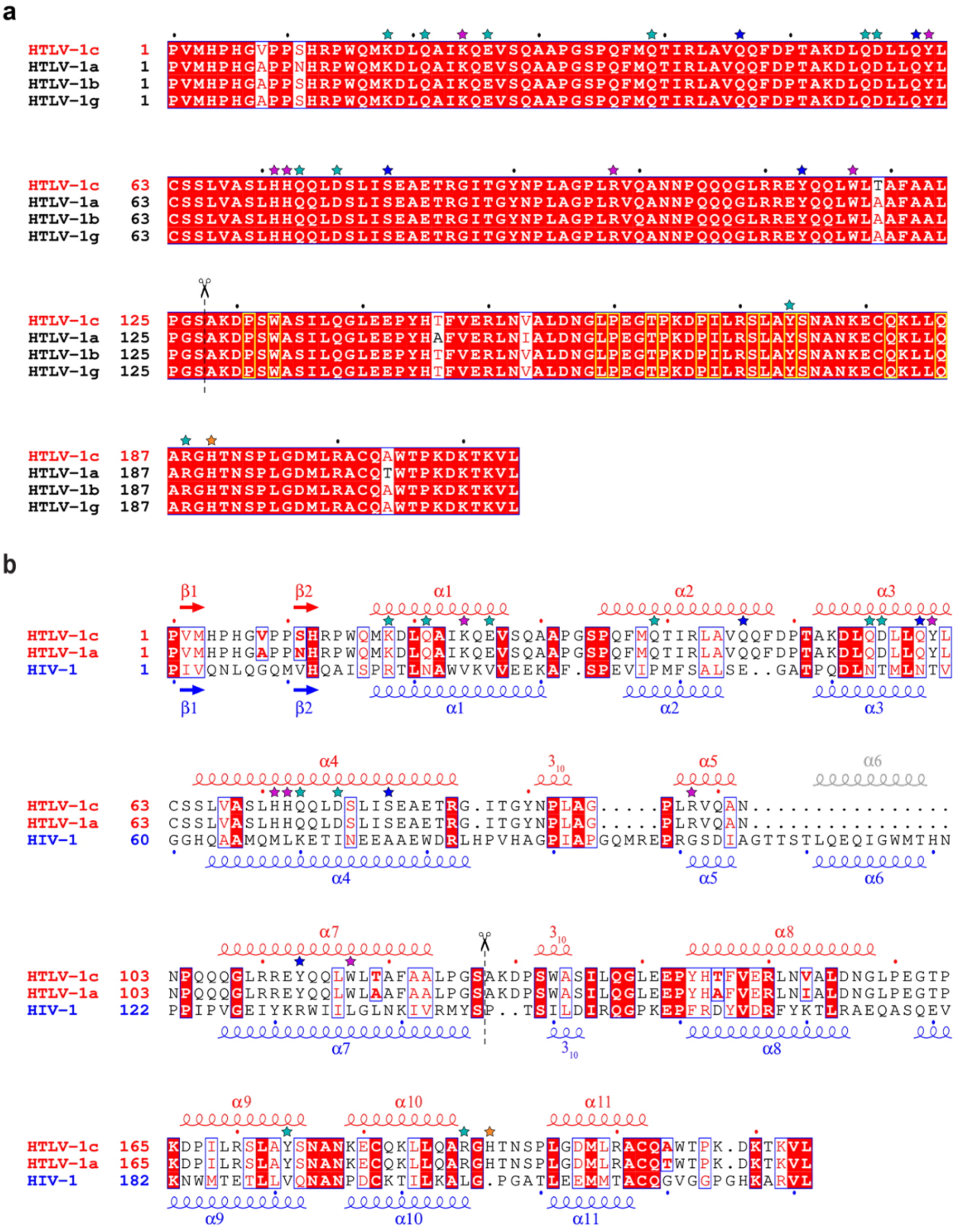

Supplementary Fig. 1. Structure-based sequence alignment of HTLV-1c, HTLV-1a and HIV-1 capsid proteins. **a**, The HTLV-1 CA sequence alignment includes subtypes a, b, c, and g. Residues involved in the CA_{CTD} dimer interface are boxed in yellow. **b**, The alignment of HTLV-1c/a CA (in red) and HIV-1 CA (in blue) showing secondary structural elements. Strictly identical residues are highlighted in red background, and the highly conserved residues are shown in red and boxed in blue. Residues are marked with stars to indicate their impact on viral infectivity and p19 production: golden stars represent increased infectivity with normal p19 production; cyan stars, decreased infectivity with normal p19 production; magenta stars, increased infectivity with reduced p19 production; and navy stars, no change in either infectivity or p19 production. The alignment figures were generated using ESPript 3[1].

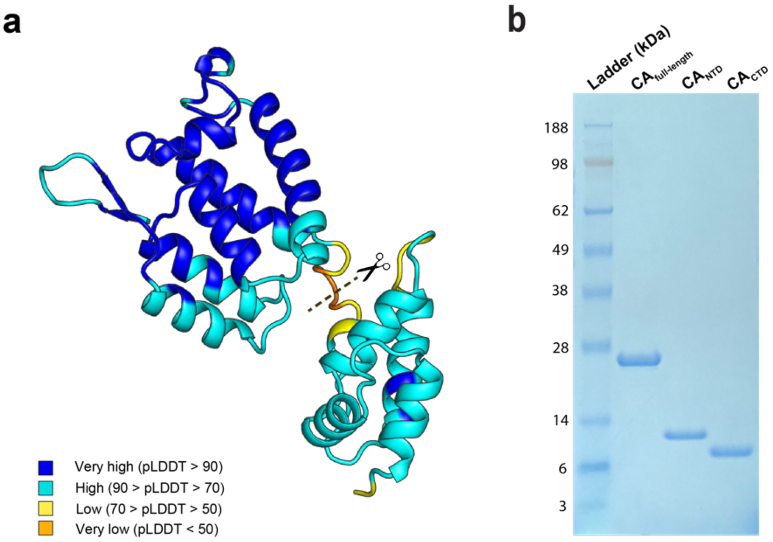

Supplementary Fig. 2. AlphaFold2-predicted model of HTLV-1 CA and SDS-PAGE analysis of CA proteins. **a**, The AlphaFold2-predicted model of HTLV-1 CA is colour-coded by confidence, as assessed by the predicted Local Distance Difference Test (pLDDT). High-confidence regions are shown in navy and cyan, while lower-confidence areas appear in yellow to orange. **b**, Purified protein samples of the full-length HTLV-1 CA, CA_{NTD}, and CA_{CTD} were separated by SDS-PAGE gel with a molecular mass ladder on the left (in kilodalton, kDa). The Uncropped SDS-PAGE gel is provided at the end of Supplementary Information.

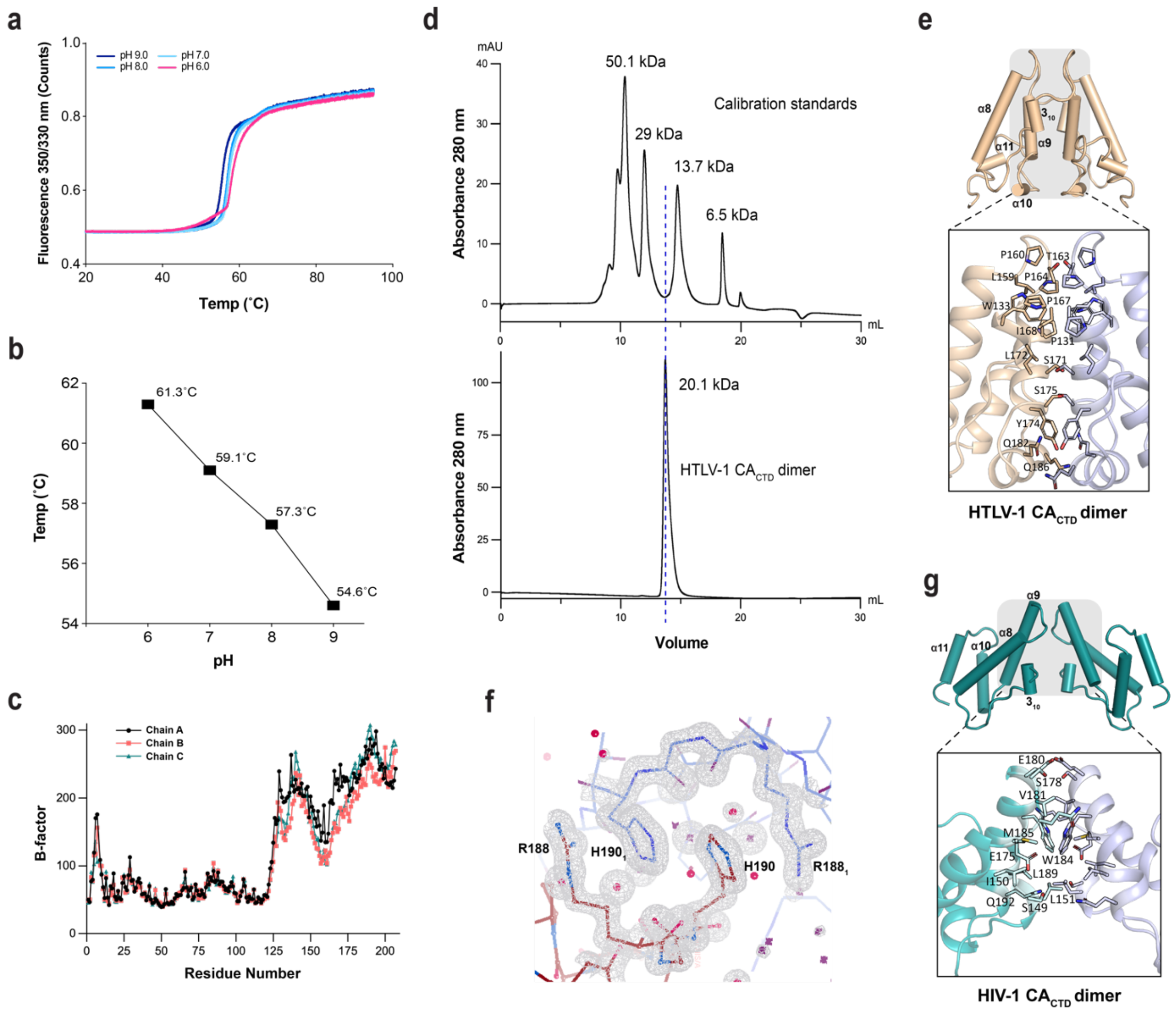

Supplementary Fig. 3. The full-length HTLV-1 CA thermal stability and CA$_{CTD}$ dimer characteristics. a, The fluorescence ratio of the full-length HTLV-1 CA measured over a temperature ramp at pH 6, 7, 8 or 9 using nanoDSF. **b**, Unfolding temperature points of the full-length HTLV-1 CA at the corresponding pH values. **c**, The structural B-factor analysis of the full-length HTLV-1 CA (NTD, residues of 1-127; CTD, residues of 128-207). The B-factor data point of each residue represents the average value of all atoms within that residue. The three traces correspond to chains A, B, and C, respectively, which belongs to an asymmetric unit. **d**, Elution curves aligned from size-exclusion chromatography display the calibration standards (top) and a dimer size of HTLV-1 CA$_{CTD}$ (bottom). **e** and **g**, Comparison of CTD dimer interfaces between HIV-1 (**e**) and HTLV-1 (**g**), with interface residues labelled. **f**, The CA$_{CTD}$ residues R188 and H190 (in red) interdigitate with R188$_1$ and H190$_1$ (in blue) from another copy of molecule generated by crystallographic symmetry. Source data are provided as a Source Data file.

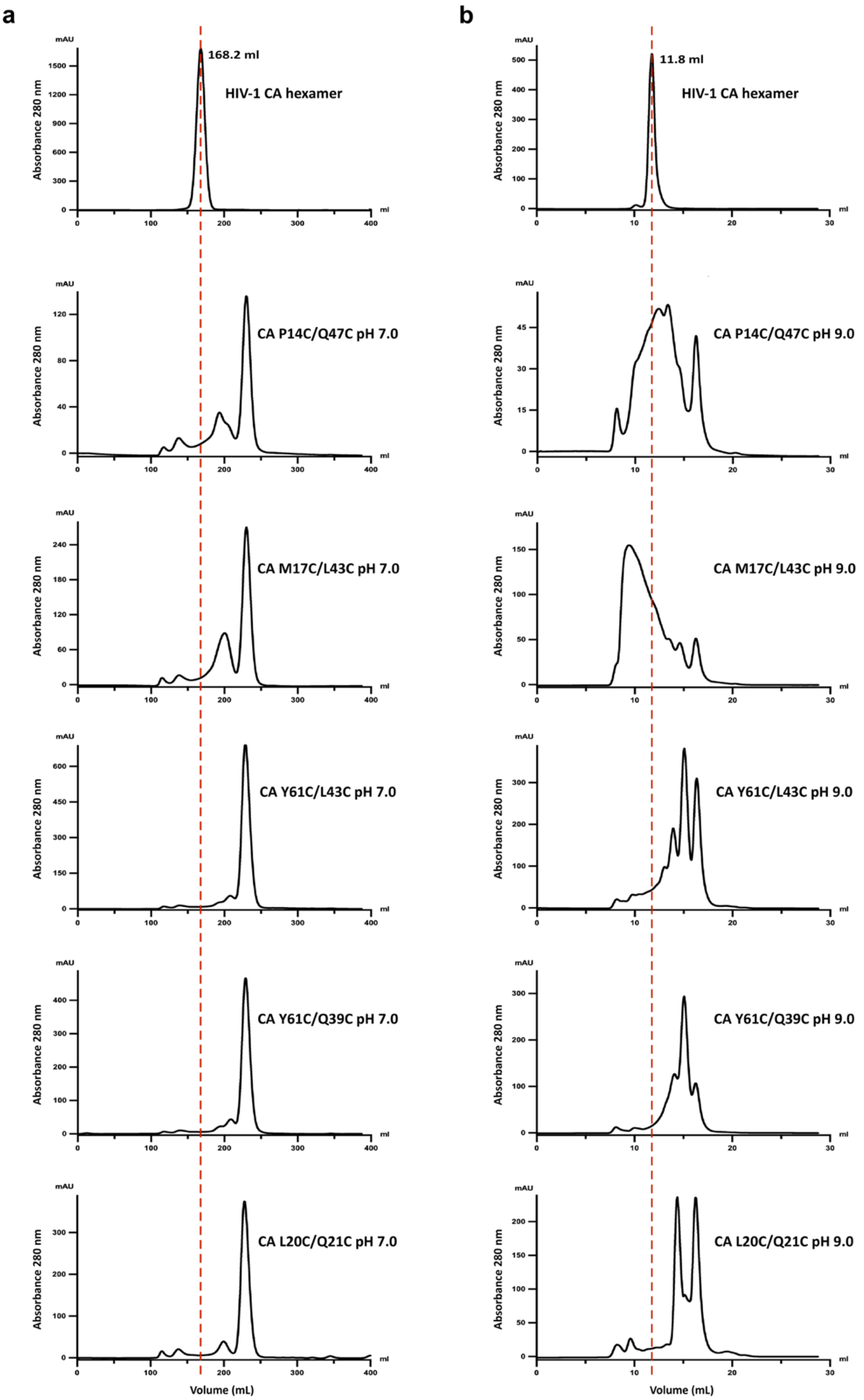

Supplementary Fig. 4. Size-exclusion chromatography of cross-linked HTLV-1 CA by cysteine substitution. a, Comparison of SEC elution peaks among the HIV-1 CA hexamer and assembled HTLV-1 CA mutants at pH 7.0 or (**b**) pH 9.0.

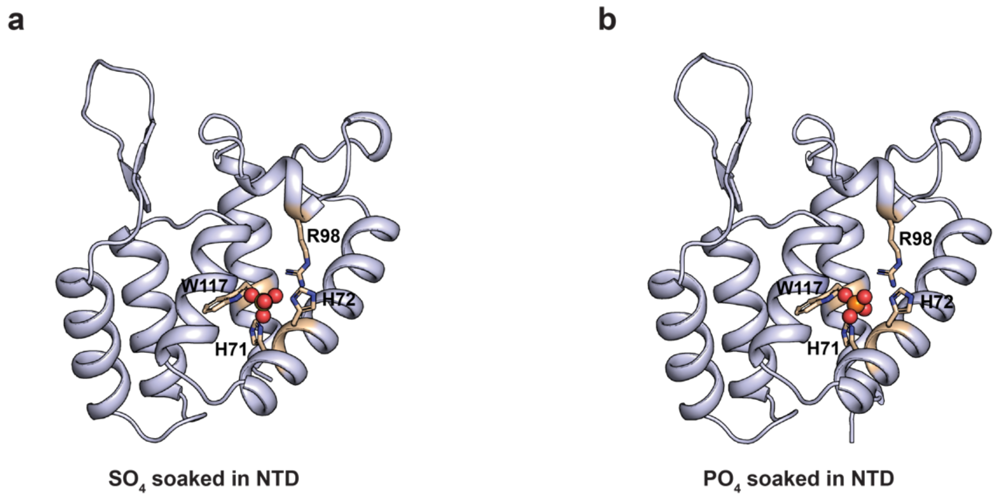

Supplementary Fig. 5. The solved sulfate-bound and phosphate-bound structures within the CA_{NTD} triclinic crystal form. A sulfate ion (**a**) or a phosphate ion (**b**) was soaked in the cross-linked CA_{NTD} triclinic crystal form, coordinating with residues H71, H72, R98 and W117.

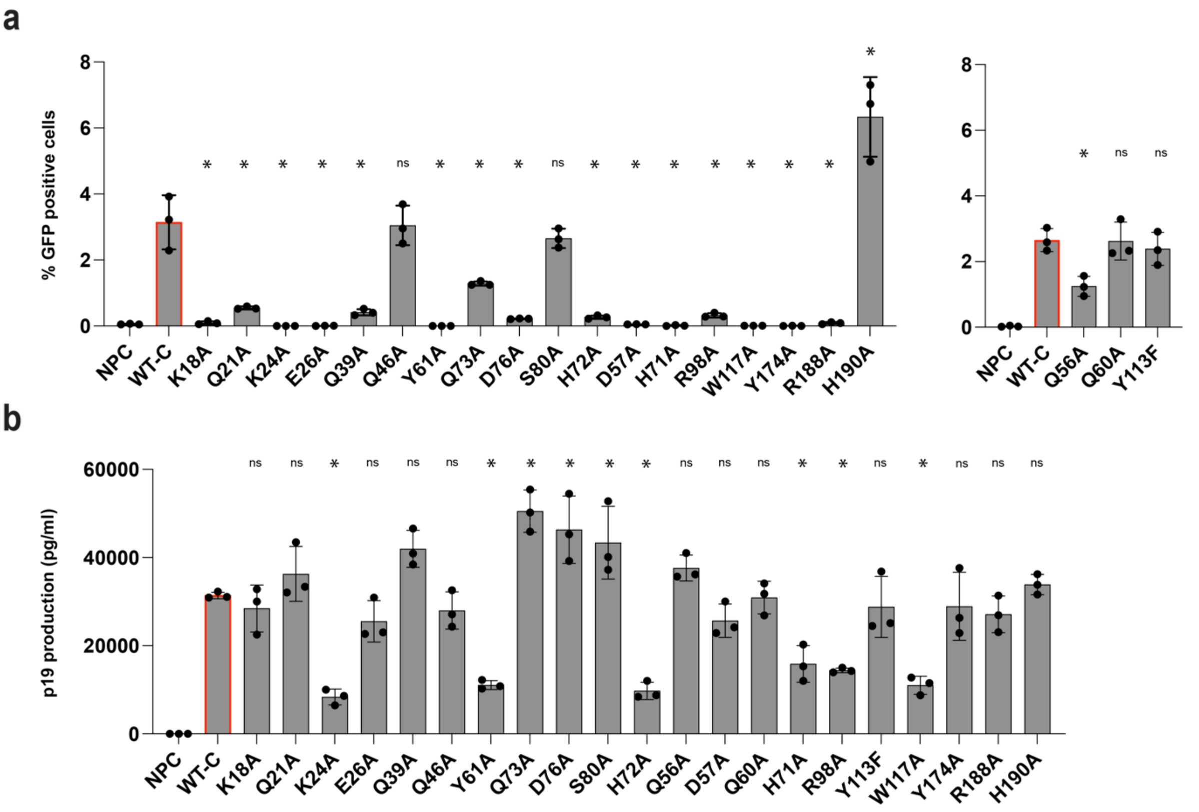

Supplementary Fig. 6. Infectivity and p19 production of HTLV-1 mutants. a, Bar graph displaying infectivity of WT-C CA mutants as measured by GFP reporter expression in a replication dependent infection system. **b**, p19 production as measured using a p19 ELISA. Results are displayed as the mean +/- SD from 3 independent experiments. Statistical analysis was performed using a one-way ANOVA with comparison to the wild-type control (WT-C, highlighted with a red border). * = $p < 0.05$, *ns* = $p > 0.05$.

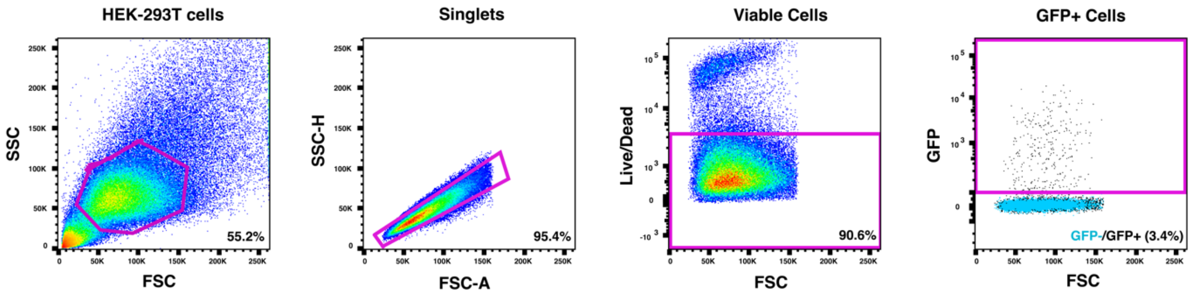

Supplementary Fig. 7. Gating strategy for the HTLV-1 infection assay. Representative flow cytometry plots outlining the gating strategy used to determine the number of infected cells (GFP+ cells) in the HTLV-1 infection assays in Fig. 6a, b, c and d.

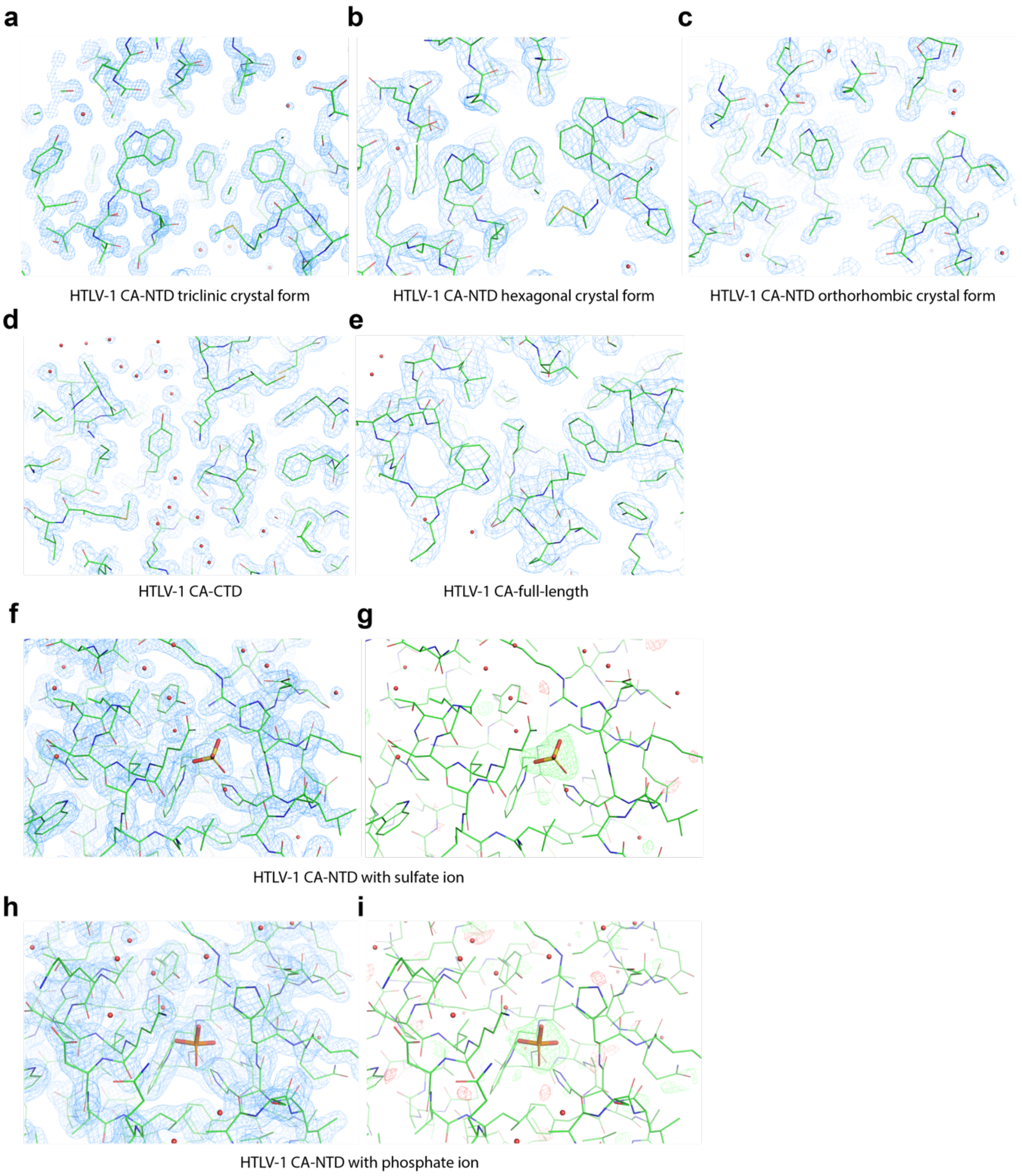

Supplementary Fig. 8. Electron density maps.
a-e, 2Fo-Fc maps of the HTLV-1 CA-NTD triclinic, hexagonal and orthorhombic crystal forms, HTLV-1 CA-CTD, and HTLV-1 CA-full-length, contoured at 1.5 σ. **f-i,** 2Fo-Fc maps contoured at 1.5 σ, and Fo-Fc omit density difference maps contoured at ± 3.0 σ for HTLV-1 CA-NTD structures with sulfate ion (**f** and **g**) or phosphate ion (**h** and **i**).

Supplementary Table 1. Data collection and refinement statistics.

	CA-full-length	NTD-triclinic	NTD-hexagonal	NTD-orthorhombic	CTD	NTD-triclinic with PO_4	NTD-triclinic with SO_4
**Data collection**							
Space group	F 2 2 2	P 1	P 6 2 2	$P\ 2_1\ 2_1\ 2_1$	$P\ 2_1\ 2_1\ 2_1$	P 1	P 1
Unit cell dimensions							
a, b, c (Å)	74.1, 128.7, 297.3	30.1, 30.6, 35.0	74.6, 74.6, 73.8	30.8, 77.3, 101.9	57.0, 94.1, 96.0	28.2, 30.7, 36.1	28.7, 30.6, 36.4
α, β, γ (°)	90, 90, 90	75.0, 68.5, 89.6	90, 90, 120	90, 90, 90	90, 90, 90	65.2, 79.3, 89.6	65.6, 70.7, 72.0
Resolution range (Å)	43.63 - 2.25 (2.33 - 2.25)	31.31 - 0.87 (0.90 - 0.87)	37.31 - 2.1 (2.18 - 2.1)	42.55 - 1.47 (1.52 - 1.47)	36.72 - 1.47 (1.52 - 1.47)	27.8 - 1.73 (1.79 - 1.73)	27.31 - 1.71 (1.77 - 1.71)
Unique reflections	33741 (3156)	84821 (7829)	7535 (722)	42342 (4151)	88358 (8662)	10884 (1071)	10368 (1005)
R-merge	0.13 (1.2)	0.05 (0.26)	0.12 (1.05)	0.07 (0.70)	0.07 (1.23)	0.06 (0.64)	0.04 (1.27)
R-meas	0.13 (1.3)	0.06 (0.30)	0.12 (1.06)	0.07 (0.73)	0.07 (1.28)	0.08 (0.77)	0.05 (1.49)
R-pim	0.04 (0.35)	0.02 (0.16)	0.02 (0.19)	0.02 (0.20)	0.02 (0.35)	0.04 (0.41)	0.03 (0.78)
I / σI	11.18 (1.45)	18.43 (4.44)	23.90 (4.78)	20.70 (3.55)	20.85 (2.11)	9.48 (1.51)	14.54 (0.92)
$CC_{1/2}$	1.0 (0.80)	1.0 (0.95)	1.0 (0.96)	1.0 (0.87)	1.0 (0.76)	1.0 (0.70)	1.0 (0.45)
Completeness (%)	99.34 (94.13)	93.00 (86.35)	99.91 (99.86)	99.92 (99.98)	99.73 (97.62)	96.87 (95.45)	91.92 (88.27)
Multiplicity	13.5 (13.0)	6.7 (3.5)	33.7 (32.5)	13.1 (13.2)	13.3 (13.2)	3.5 (3.4)	3.6 (3.5)
**Refinement**							
Resolution (Å)	43.63 - 2.25	31.31 - 0.87	48.61 - 2.1	42.55 - 1.47	36.72 - 1.47	27.8 - 1.73	32.22 - 1.71
Reflections used in refinement	33695 (3128)	84807 (7829)	7533 (721)	42327 (4150)	88201 (8520)	10883 (1071)	10336 (978)
Reflections used for R-free	1771 (168)	4178 (375)	738 (68)	4297 (434)	4385 (441)	538 (47)	1035 (98)
R_{work}/ R_{free}	0.251/ 0.288	0.120/ 0.131	0.240/ 0.270	0.182/ 0.207	0.168/ 0.193	0.200/ 0.230	0.201/ 0.250
Number of atoms	4927	1240	1009	2234	4419	1049	1021
Protein	4830	1081	984	2010	3860	985	965
Ion	0	0	5	5	35	5	5
Water	97	159	20	219	524	59	51
Wilson B-factor ($Å^2$)	43.24	8.41	32.79	15.95	19.29	28.81	29.06
Protein	108.53	9.78	39.46	21.54	26.96	35.5	33.51
Ion			37	14.51	30.95	35.41	28.56
Water	57.86	20.67	37.81	28.69	34.77	39.07	38.14
R.M.S deviations							
Bond lengths (Å)	0.004	0.009	0.003	0.004	0.007	0.003	0.003
Angles (°)	0.65	1.13	0.51	0.7	0.88	0.59	0.54
Ramachandran outliers (%)	0	0	0	0	0	0	0
Ramachandran favored (%)	95.38	99.2	99.17	98.8	98.53	98.37	99.17
**PDB ID**	8ERI	8ERE	8ERG	8ERF	8ERH	8TMV	8TMW

Statistics for the highest-resolution shell are shown in parentheses.

Supplementary Table 2. Primer sequences used for generating HTLV-1c CA mutants.

CA Mutant	Primer Sequence (5'–3')
K18A FW	CGTGGCAAATGGCGGACCTACAGG
K18A RV	CCTGTAGGTCCGCCATTTGCCACG
Q21A FW	ATGAAGGACCTAGCGGCCATCAAGC
Q21A RV	GCTTGATGGCCGCTAGGTCCTTCAT
K24A FW	CTACAGGCCATCGCGCAAGAGGTCTC
K24A RV	GAGACCTCTTGCGCGATGGCCTGTAG
E26A FW	CATCAAGCAAGCGGTCTCCCAAG
E26A RV	CTTGGGAGACCGCTTGCTTGATG
Q39A FW	CCCAGTTTATGGCGACCATCCGGC
Q39A RV	GCCGGATGGTCGCCATAAACTGGG
Q46A FW	GGCTTGCAGTGGCGCAGTTTGACC
Q46A RV	GGTCAAACTGCGCCACTGCAAGCC
Q56A FW	TGCCAAGGACCTCGCGGACCTCCTGCAGTA
Q56A RV	TACTGCAGGAGGTCCGCGAGGTCCTTGGCA
D57A FW	GGACCTCCAGGCCCTCCTGCAGTAC
D57A RV	GTACTGCAGGAGGGCCTGGAGGTCC
Q60A FW	CAGGACCTCCTGGCGTACCTTTGCTC
Q60A RV	GAGCAAAGGTACGCCAGGAGGTCCTG
Y61A FW	GACCTCCTGCAGGCCCTTTGCTCCTC
Y61A RV	GAGGAGCAAAGGGCCTGCAGGAGGTC
H71A FW	TAGCCTCCCTCGCTCATCAGCAGC
H71A RV	GCTGCTGATGAGCGAGGGAGGCTA
H72A FW	CTCCCTCCATGCTCAGCAGCTAG
H72A RV	CTAGCTGCTGAGCATGGAGGGAG
Q73A FW	CCCTCCATCATGCGCAGCTAGATAG
Q73A RV	CTATCTAGCTGCGCATGATGGAGGG
D76A FW	CATCAGCAGCTAGCTAGCCTTATATC
D76A RV	GATATAAGGCTAGCTAGCTGCTGATG
S80A FW	GATAGCCTTATAGCTGAGGCTGAG
S80A RV	CTCAGCCTCAGCTATAAGGCTATC
R98A FW	CCGGTCCCCTCGCTGTCCAAGCCAAC
R98A RV	GTTGGCTTGGACAGCGAGGGGACCGG
Y113F FW	GAGGCGAGAATTCCAGCAACTTTG
Y113F RV	CAAAGTTGCTGGAATTCTCGCCTC
W117A FW	TACCAGCAACTTGCGCTCACCGCATTTG
W117A RV	CAAATGCGGTGAGCGCAAGTTGCTGGTA
Y174A FW	GCTCCTTAGCCGCCTCCAATGCAAAC
Y174A RV	GTTTGCATTGGAGGCGGCTAAGGAGC
R188A FW	TACTACAGGCCGCAGGACACACCAATAG
R188A RV	CTATTGGTGTGTCCTGCGGCCTGTAGTA
H190A FW	AGGCCCGAGGAGCCACCAATAGCC
H190A RV	GGCTATTGGTGGCTCCTCGGGCCT

FW and RV stand for forward and reverse, respectively.

Uncropped Coomassie Blue stained SDS-PAGE in the Supplementary Fig. 2b

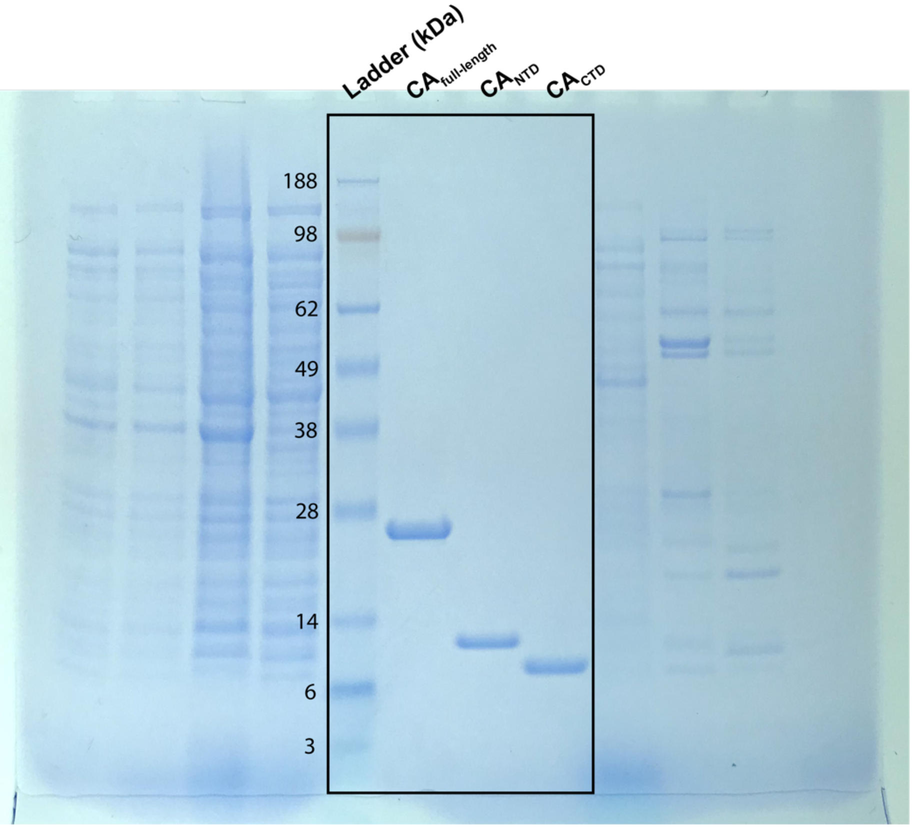

Supplementary Reference

1. Robert, X. & Gouet, P. Deciphering key features in protein structures with the new ENDscript server. *Nucleic Acids Res.* **42**, W320–W324 (2014).
