# Supplementary material for: The Human T-cell Leukemia Virus capsid protein is a potential drug target
Source: Nat Commun. 2025 Dec 4;16:10892. doi: 10.1038/s41467-025-65899-2 (PMC12678768; doi:10.1038/s41467-025-65899-2)
Supplement: Supplementary file 3 — Description of Additional Supplementary Files [file 41467_2025_65899_MOESM3_ESM.pdf]

Description of Additional Supplementary Files

File: Supplementary Movie 1

Description: The sulfate binding site on the HTLV-1 CA-NTD. Structural morph between apo and sulfate-bound states of HTLV-1c CA NTD. Residues Q60, H71, H72, R98, and W117 are shown as sticks. The sidechains of residues Q56, H72 and R98 flip up and down to allow the sulfate entry into the pocket
